# SUOH 03 Guidewire for the Management of Coronary Artery Dissection: Insights from a Multicenter Registry

**DOI:** 10.1155/2023/7958808

**Published:** 2023-08-01

**Authors:** Gabriele L. Gasparini, Mario Bollati, Mauro Chiarito, Michele Cacia, Fausto Roccasalva, Claudiu Ungureanu, Giuseppe Colletti, Simone Muraglia, Pierluigi Merella, Fabrizio Ugo, Andrea Pacchioni, Salvatore Colangelo, Jorge Sanz Sanchez, Pier Pasquale Leone, Azeem Latib, Pietro Mazzarotto

**Affiliations:** ^1^IRCCS Humanitas Research Hospital, Rozzano, Italy; ^2^Ospedaliero di Lodi, Lodi, Italy; ^3^Department of Biomedical Sciences, Humanitas University, Pieve Emanuele, Milan, Italy; ^4^Hôpital de Jolimont, La Louvière, Belgium; ^5^Clinique Saint-Joseph, Arlon, Belgium; ^6^Ospedale Santa Chiara, Trento, Italy; ^7^Azienda Ospedaliero Universitaria di Sassari, Sassari, Italy; ^8^Sant' Andrea Hospital, Vercelli, Italy; ^9^Ospedale di Mirano, Mirano, Italy; ^10^Ospedale San Giovanni Bosco, Turin, Italy; ^11^Hospital Universitari i Politècnic La Fe, Valencia, Spain; ^12^Centro de Investicacion Biomedica en Red Efermedades Coardiovasculares (CIBERCV), Madrid, Spain; ^13^Montefiore Health System, Bronx, New York City, NY, USA

## Abstract

**Background:**

In the setting of coronary artery dissection, both spontaneous and iatrogenic, fixing the intimal tear, usually with stent implantation, can be extremely challenging if the distal wire position has been lost. Common complications are mainly related to the inadvertent subintimal tracking of the guidewire while attempting to gain the distal true lumen.

**Aims:**

To report the registry results of using the SUOH 0.3 guidewire for managing coronary artery dissection in a real-world multicenter setting.

**Methods:**

The study population in this retrospective, multicenter, international registry included 75 consecutive patients who underwent PCI and required an antegrade wiring of a dissected coronary artery.

**Results:**

Successful use of SUOH 0.3 was achieved in 69 (92%) patients. The use of a microcatheter was associated with a significantly higher rate of TIMI 3 flow at the end of the procedure (no microcatheter: *n* = 17, 81%; microcatheter: *n* = 52, 96.3%; *p* = 0.017). The first recanalization attempt was made with the SUOH 03 guidewire in 48 (64%) cases, and it was successful in 42 (87%). The overall PCI success rate was reported in 72 (96%) patients, with no significant differences among patients with different origins, mechanisms, and locations of dissection.

**Conclusions:**

In this setting, the SUOH 0.3 guidewire provides high procedural success without additional complex techniques.

## 1. Introduction

Arterial wall dissection results from mechanical disruption of the endothelium, blood extravasation into the subendothelial space, and subsequent false lumen creation within the tunica media. The false lumen may compress the true lumen to total occlusion, leading to flow impairment, tissue hypoperfusion, and necrosis [1]. Coronary artery dissection may be spontaneous, traumatic, or iatrogenic [2–4]. Spontaneous coronary artery dissection (SCAD) is a rare but potentially life-threatening event, predominantly seen in the young female population, especially postpartum. The optimal treatment strategy for patients with SCAD is still unclear but is likely based on several clinical and angiographic factors [2]. Iatrogenic coronary artery dissection (ICAD) during coronary angiography or percutaneous coronary intervention (PCI) can be caused by a variety of mechanisms; the most frequent include guide-catheter-induced ostial injury, balloon dilation, aggressive wire manipulation, and the use of atherectomy devices [1, 4, 5]. Fixing the intimal tear and restoring vessel flow, usually with stent implantation, is easily achieved if a guidewire is already present in the distal true lumen, but it can be extremely challenging if the distal wire position has been lost. One of the main reasons for procedural failure during PCI for spontaneous and iatrogenic coronary artery dissections is the difficulty of advancing a guidewire into the distal true lumen. Limited data are available in the published literature regarding the structural properties of guidewires and the manipulation techniques required to maximize the chances of successful coronary wiring. In a previously published hypothesis-generating case series, the results of using the SUOH 0.3 “rope coil” wire (Asahi Intecc, Japan) for coronary dissection tracking were reported [6]. In addition, the “soft wire technique” was described as an effective strategy to regain the true lumen after coronary artery dissection, either before wiring or after wire loss. To our knowledge, this is the only attempt to define the optimal structural properties and necessary manipulation techniques of a specific guidewire for managing coronary artery dissection. The present investigation aims to report the results of using the SUOH 0.3 guidewire for managing coronary artery dissection in a real-world multicenter registry.

## 2. Methods

### 2.1. Study Design

The study population in this retrospective, multicenter, international registry included all consecutive patients from March 2020 to October 2022 in 10 high-volume centers who underwent PCI and in whom antegrade wiring of a dissected coronary artery was clinically required for either spontaneous or iatrogenic dissection. The inclusion criteria were broad and reflected routine clinical practice, including patients with stable CAD and acute coronary syndromes. No limits were set for the number of treated lesions, vessels, or lesion length, and no patients were excluded based on comorbid conditions or age. PCI was performed according to standard techniques, and the devices used were left to operator preference. Each patient provided informed consent for participation in the study. This study complies with the Declaration of Helsinki and was approved by the local Ethics Committee.

### 2.2. Device Description

**Figure 1:**
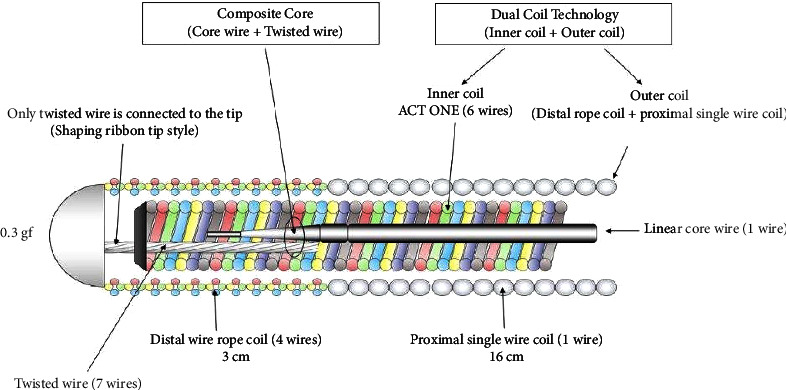
Technical characteristics of the SUOH 0.3 guidewire. Readapted from Gasparini et al. Catheter Cardiovasc Interv 96, E462–E466 (2020) [6].

The SUOH 0.3 is a composite core, dual coil (Sion Tecc, Asahi Intecc Co, Japan), 0.014″, flat tip guidewire originally developed for tortuous collateral channel tracking. The “composite core” technology consists of combining a classic linear core wire with a second twisted wire in parallel, conjoined at the tip. The result is increased and finer torque control (1 : 1) of the tip. “Dual coil technology” consists of a second intertwined inner rope coil, the so-called “ACT ONE,” within the classic outer coil allowing optimal and durable shaping of the tip of the wire with improved directional control. This wire has a unique combination of technical characteristics: (1) the distal 3 cm of the outer “rope coil” consists of 4 intertwined wires instead of one, resulting in higher tip flexibility, while the proximal 16 cm is made of a conventional single wire rope coil; (2) only the twisted wire of the composite core is connected to the tip in a shaping ribbon tip style, providing tip shape retention and softness; (3) the dual twisted coil structure allows high torqueability and steerability while maintaining good support; and (4) the distal 1 mm tip is preshaped to a 45° angle offering excellent shape memory and retention, with the softest tip load (0.3 gf) among available contemporary guidewires (Figure 1).

### 2.3. Definitions and Endpoints

Spontaneous coronary artery dissection was defined as a nontraumatic and noniatrogenic tear in one of the epicardial coronary arteries. Iatrogenic coronary dissection was defined as angiographic evidence of an intimal tear secondary to guide-catheter coronary ostia engagement, forceful injection of contrast medium, wire manipulation, balloon dilation, and use, passage, or deployment of any other interventional device with consequent wire positioning loss. The grading of dissections was described according to National Heart, Lung, Blood Institute (NHLBI) classification: [7].

 
*Type A*. Minor radiolucent areas in the lumen without impairment of flow or persistent dye staining after contrast run-off
 
*Type B*. Luminal flap that is radiolucent and runs parallel to the vessel wall with contrast injection but without impairment of flow or persistent dye staining after contrast run-off
 
*Type C*. Contrast appears outside of the vessel lumen as an “extraluminal cap” and the staining appears even after contrast clears off the lumen
 
*Type D*. Spiral radiolucent luminal filling defects, often persistent staining after contrast clears from the vessel
 
*Type E.* New and persistent filling defects in the vessel lumen.
 
*Type F*. Lesions that progress to impaired flow or total occlusion.

The primary endpoint was the successful use of a SUOH 0.3 guidewire, defined as the gain of the true lumen with the guidewire. The key secondary endpoints were PCI success, defined as achievement of TIMI ≥2 flow at the end of the procedure, and successful use of a SUOH 0.3 guidewire at the first attempt.

### 2.4. Statistical Analysis

Statistical analysis was performed using Stata (version 16.0, Stata Corp., College Station, Texas, USA). Continuous variables are reported as the mean ± standard deviation (SD) or median and interquartile range and were compared with Student's *t*-test or Mann–Whitney or Wilcoxon tests with the normality of the data verified by the Kolmogorov–Smirnov goodness-of-fit test. Categorical variables are reported as *N* (%) and were compared with the *χ*2 test with Yates correction for continuity or the Fisher exact test as appropriate. Two-sided *p* values < 0.05 were considered statistically significant.

## 3. Results

### 3.1. Baseline Clinical and Procedural Characteristics

**Figure 2:**
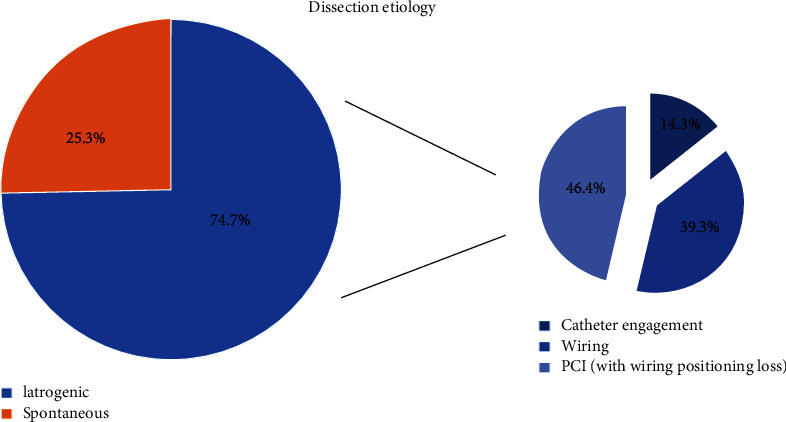
Dissection etiology in the overall cohort.

**Figure 3:**
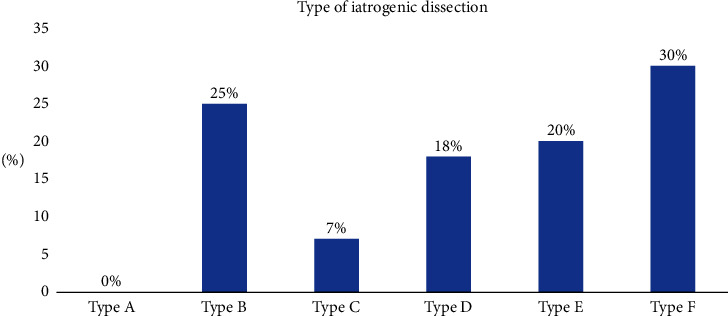
Types of iatrogenic dissection in the overall cohort. Type A minor radiolucent areas in the lumen without impairment of flow or persistent dye staining after contrast run-off; type B luminal flap that is radiolucent and runs parallel to the vessel wall with contrast injection, but without impairment of flow or persistent dye staining after contrast run-off; type C contrast appears outside the vessel lumen as an “extraluminal cap,” and the staining appears even after contrast clears off the lumen; type D spiral radiolucent luminal filling defects, often persistent staining after contrast clears off the lumen; type E new and persistent filling defects in the lumen; type F lesions that progress to impaired flow or total occlusion.

Baseline clinical data are reported in Table 1. A total of 75 patients with coronary dissection were included in the present analysis. The mean age was 64.1 ± 13.6 years, and the female gender was predominant (56% of cases). Cardiovascular risk factors were prevalent, with hypertension and dyslipidemia reported in around 70% of patients. Diabetes was present in one-third of cases. Illicit drug use was rare (less than 2%). The clinical indication for coronary angiography was acute myocardial infarction in more than half of all cases (NSTEMI: *n* = 26, 34.7%; STEMI: *n* = 15, 20%). No clinical or angiographic differences were detected depending on dissection etiology although iatrogenic dissection was more common (*n* = 56, 75%); half of the iatrogenic cases occurred during wiring or predilatation (Figure 2, Table 2). Ostial catheter dissection occurred in 8 patients (14%). Dissection distribution was uniform between the left anterior descending, circumflex, and right coronary arteries, while 6.7% of dissections involved the left main coronary artery. The proximal, middle, and distal segments were equally affected by dissection. Flow impairment was present in 49 patients (71%), leading to hemodynamic instability in 14 patients (19%). Even in iatrogenic dissection, vessel occlusion was observed in one-third of cases (“type F″ dissection, Figure 3). No significant differences were observed in TIMI flow among spontaneous and iatrogenic dissections: TIMI flow <2 was reported in 32 patients with spontaneous dissection and 13 with iatrogenic dissection (57% vs. 68%, *p* = 0.63).

### 3.2. Angiographic and Procedural Outcomes

**Figure 4:**
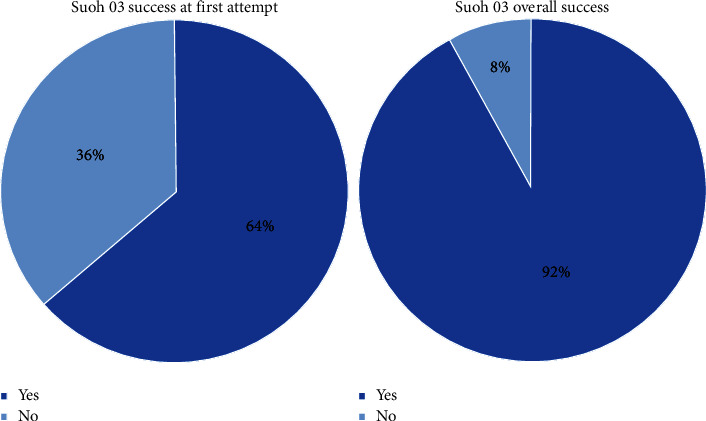
SUOH 0.3 success rate at first attempt (a) and overall (b).

The primary endpoint, successful use of a SUOH 0.3 guidewire, was achieved in 69 (92%) patients (Figure 4). No significant correlation was found with the use of a microcatheter, which was reported in 72% of cases. Conversely, the use of a microcatheter was associated with a significantly higher rate of TIMI 3 flow at the end of the procedure (no microcatheter: *n* = 17, 81%; microcatheter: *n* = 52, 96.3%; *p* = 0.017). In 64% of cases, the first recanalization attempt was made with SUOH 03 guidewire. Successful use of SUOH 0.3 at first attempt was achieved in 42 (87%) patients. Overall PCI success was reported in 72 (96%) patients, with no significant differences among patients with different origins, mechanisms, and locations of dissection. Among patients presenting with TIMI flow 0 (*n* = 22), successful use of the SUOH 0.3 guidewire was reported in 18 (82%) patients, and PCI success was achieved in 20 (91%) patients. Among patients presenting with type F dissections (*n* = 17), 88% (*n* = 15) of patients had successful use of SUOH 0.3, and PCI success was 94% (*n* = 16). Intraprocedural IVUS evaluation was performed in 18 (24%) patients.

## 4. Discussion

Coronary artery dissection treatment remains a major issue. Gaining the distal true lumen with the guidewire is the most challenging step when indicated in SCAD or in cases of ICAD when the distal wire position has been lost. In this context, intravascular ultrasound (IVUS) can visualize the dissection entry point, confirm the presence of the wire in the true lumen, and provide useful information regarding dissection length and vessel diameter that can guide stent selection. However, this maneuver carries potential risks, such as extending the coronary dissection with the imaging catheter. Optical coherence tomography imaging, although previously described, is not recommended due to the need for contrast injection. If antegrade wiring cannot be readily accomplished, alternative techniques derived from chronic total occlusion (CTO) interventions to gain distal true lumen have been described, including subintimal tracking and reentry (STAR), antegrade fenestration and reentry (AFR), limited antegrade subintimal tracking (LAST), subintimal reentry using the Stingray balloon, and whenever possible retrograde recanalization through collateral channels [8, 9]. However, some of the techniques mentioned above are time-consuming, others require dedicated devices (i.e., the stingray system), and all are intended for experienced and CTO-PCI-trained operators. Moreover, IVUS-guided subintimal reentry, also described as a possible technique during acute vessel closure, requires significant expertise in IVUS interpretation. Given the high complexity of the technique and the possibility of widening the antegrade dissection, it should only be considered a “bailout” procedure. Antegrade wiring of the true lumen should be the first-line strategy in patients presenting with coronary artery dissection. Crossing or recrossing the dissected segment can be accomplished with a soft workhorse guidewire with a low tip load. Soft workhorse guidewires provide enhanced tactile and visual feedback, increasing the chance of successfully finding the true lumen. It is imperative to avoid creating new dissection planes or causing acute vessel occlusion when attempting to gain wire access into the true lumen. Limited data are available regarding the structural properties of the guidewires and the manipulation techniques to maximize the chance of success. Among the wide range of available coronary guidewires, the SUOH 03 guidewire has certain structural features that make it ideal for gaining the true lumen into, or distally to, the dissected segment, thereby minimizing the risk of enlargement and extension of the dissection. The shaping ribbon tip style, the hydrophilic coating, and the soft and low-weight tip load combined with the “rope coil” technology ensure a nontraumatic advancement, high torque control, and durable preservation of the tip shape. Observing the advancement of the guidewire is crucial in discriminating between endoluminal and subintimal tracking, therefore limiting the propagation of dissection during advancement into the diseased segment. The tip curling in the subintimal cul-de-sac is an immediate sign of subintimal tracking, while wire progression without tip deflection (“straight tip progression”) stands for true lumen tracking [6]. Such visual feedback combined with high directional control allows repeating gentle and nontraumatic attempts to move from the false into the true lumen, with no need for contrast dye injections. Moreover, we observed that a guidewire's ability to facilitate spontaneous intraluminal tracking during cardiac motion is mainly related to tip flexibility. In case of tortuosity, the use of a microcatheter improves the wire control and allows confirmation of endoluminal position with blood backflow. In this multicenter registry, we analyzed 75 consecutive cases of coronary artery dissections, both ICAD and SCAD, treated with PCI in 10 mid-to-high-volume centers worldwide. Our data reveal a high success rate (92%), which is not comparable with other results due to the paucity of published data in such clinical scenarios. In their 302 consecutive SCAD cases, Mori et al. reported 100 patients who underwent *ad hoc* PCI (33.1%), 78% of which were classified as successful [10]. Common complications included false lumen stenting and proximal and distal dissection propagation, mainly related to the inadvertent subintimal tracking of the guidewire during the attempts to gain the distal true lumen [10]. In our series, the high success rate is constant between ICAD and SCAD, irrespective of dissection mechanism or location. This result is even more relevant considering that dissections in proximal or mid-coronary segments were about 62%, and the prevalence of flow impairment was 71%, two elements with potential relevant clinical implications in case of unsuccessful treatment. In tortuous vessels, using a microcatheter results in better wire control, reducing hematoma enlargement and increasing the chance of gaining the distal true lumen. In our study, microcatheter used to tackle tortuosity and improve wire control was the only predictor of final TIMI III flow. No variability between the participating centers has been revealed, supporting the reproducibility and predictability of this technique.

## 5. Conclusions

Despite the potentially unfavorable clinical impact of coronary artery dissection and the need for complicated treatments, the “soft tip wire technique” based on using the SUOH 0.3 guidewire has proven to provide high procedural success without the need for additional complex techniques. Larger studies, including results from non-CTO PCI centers, would further clarify the role of this technique in the treatment of coronary artery dissection.

## Figures and Tables

**Table 1:** Baseline clinical characteristics.

Age, years ± SD	64.1 ± 13.6
Female, *N* (%)	42 (56)
Diabetes, *N* (%)	21 (30)
Hypertension, *N* (%)	51 (69.9)
Dyslipidemia, *N* (%)	52 (73.2)
Active smoker, *N* (%)	21 (30)
Illicit drug, *N* (%)	1 (1.5)

*Clinical indication*	
Elective, *N* (%)	34 (45.3)
NSTEMI, *N* (%)	26 (34.7)
STEMI, *N* (%)	15 (20)

SD: standard deviation; NSTEMI:  non-ST elevation myocardial infarction; STEMI: ST elevation myocardial infarction.

**Table 2:** Procedural characteristics.

Dissection etiology	
Iatrogenic, *n* (%)	56 (74.7)
Spontaneous, *n* (%)	19 (25.3)
Iatrogenic dissection mechanism, *n* (%)	
Catheter engagement, *n* (%)	8 (14.3)
Wiring, *n* (%)	22 (39.3)
PCI (with wiring positioning loss)	26 (46.4)
Iatrogenic dissection classification	
Type A, *n* (%)	0 (0)
Type B, *n* (%)	14 (25)
Type C, *n* (%)	4 (7.1)
Type D, *n* (%)	10 (17.9)
Type E, *n* (%)	11 (19.6)
Type F, *n* (%)	17 (30.4)
SCAD classification	
Double lumen or luminal flap, *n* (%)	25 (44.6)
Long smooth tapering, *n* (%)	22 (39.3)
Focal stenosis, *n* (%)	9 (16.1)
Flow limiting	
No, *n* (%)	20 (29)
Yes, *n* (%)	49 (71)
TIMI flow	
0, *n* (%)	22 (29.3)
1, *n* (%)	23 (30.7)
2, *n* (%)	14 (18.7)
3, *n* (%)	16 (21.3)
Location vessel	
LM, *n* (%)	5 (6.7)
LAD, *n* (%)	23 (30.6)
CX, *n* (%)	21 (28)
RCA, *n* (%)	26 (34.7)
Segment location	
Proximal, *n* (%)	19 (25.3)
Mid, *n* (%)	28 (37.3)
Distal, *n* (%)	28 (37.4)
Hemodynamic instability	
No, *n* (%)	61 (81.3)
Yes, *n* (%)	14 (18.7)
Ongoing ischemia at angio	
No, *n* (%)	19 (25.3)
Yes, *n* (%)	56 (74.7)
SUOH 03 at first attempt	
No, *n* (%)	27 (36)
Yes, *n* (%)	48 (64)
Microcatheter support	
No, *n* (%)	21 (28)
Yes, *n* (%)	54 (72)
Suoh03 success	
No, *n* (%)	6 (8)
Yes, *n* (%)	69 (92)
PCI success	
No, *n* (%)	3 (4)
Yes, *n* (%)	72 (96)
Final TIMI	
1, *n* (%)	3 (4)
2, *n* (%)	3 (4)
3, *n* (%)	69 (92)

SCAD: spontaneous coronary artery dissection; TIMI: thrombolysis in myocardial infarction; PCI: percutaneous coronary intervention; LM: left main; LAD: left anterior descending; CX: circumflex; RCA: right coronary artery.

## Data Availability

Underlying data are not publicly available due to concerns for patient privacy and commercial confidentiality.

## Additional Points

The absence of a control group precludes a direct comparison with unselected workhorse guidewires. The lack of a prespecified CAD PCI protocol may represent an important source of bias. Moreover, all the participating centers have an active and robust CTO program, with related complex PCI skills and mid-to-high procedure volumes. These elements may represent bias due to the operators' CTO and complex PCI skills and their previous experience using the SUOH 0.3 guidewire in complex settings other than CAD.
